# High-Grade Proteus mirabilis Bacteremia of Unclear Source Complicated by Infective Endocarditis and Embolic Stroke

**DOI:** 10.7759/cureus.112336

**Published:** 2026-07-09

**Authors:** Maria Yurina, Bethel Shiferaw

**Affiliations:** 1 Hospital Medicine, Yale New Haven Hospital, New Haven, USA; 2 Internal Medicine, Saint Mary's Hospital, Waterbury, USA

**Keywords:** bacteremia, culture negative endocarditis, embolic stroke, infective endocarditis, native valve endocarditis, non hacek gram negative endocarditis, proteus mirabilis, subacute endocarditis

## Abstract

Infective endocarditis (IE) due to *Proteus mirabilis* is rare, particularly in the absence of an identifiable primary source. We report a case of a woman in her 50s with a history of remote IE and polysubstance use disorder who presented with progressive gait slowing and weakness over one month. Neuroimaging revealed an acute-to-subacute infarct in the right anterior cerebral artery territory.

Further evaluation demonstrated mitral valve abnormalities with suspected vegetation, and subsequent blood cultures grew *Proteus mirabilis* in all four blood culture bottles. Notably, the initial blood cultures obtained during admission were negative despite subsequent high-grade bacteremia. Extensive evaluation failed to identify a primary genitourinary, gastrointestinal, or respiratory source of bacteremia.

The patient was treated with a six-week course of IV ceftriaxone, resulting in microbiologic clearance and clinical improvement, with a residual mild neurologic deficit. Follow-up echocardiography demonstrated persistent valvular abnormalities, likely representing fibrotic sequelae.

This case highlights a rare etiology of native valve endocarditis, the diagnostic challenges of subacute presentations, and the importance of comprehensive investigation in patients with stroke of unclear etiology.

## Introduction

Infective endocarditis (IE) due to *Proteus mirabilis* is rare and is typically associated with genitourinary infections or underlying structural abnormalities. Within the broader spectrum of non-HACEK Gram-negative IE, *Proteus* species represent an exceptionally infrequent cause of native valve involvement, particularly in the absence of an identifiable infectious source.

Non-HACEK Gram-negative IE is uncommon but is associated with delayed diagnosis, higher morbidity, and an increased risk of embolic complications [1,2]. *Proteus mirabilis* is a particularly rare cause of native valve endocarditis, with most reported cases linked to genitourinary sources or structural abnormalities [3].

We report a case of *Proteus mirabilis* bacteremia of unclear source complicated by native valve IE and ischemic stroke, most likely secondary to septic embolism.

## Case presentation

A woman in her 50s presented to the ED with approximately one month of progressive gait slowing and generalized weakness, without a sudden focal neurologic deficit. Her daughter noted progressively slowed ambulation, prompting evaluation. The patient denied acute limb weakness or numbness at presentation.

She had an extensive medical history, including opioid use disorder on methadone maintenance therapy, a history of cocaine use, recently treated hepatitis C virus infection, and a remote history of IE. According to the patient, this episode occurred many years before the current presentation; however, the causative organism, valve involved, treatment received, and any residual valvular abnormalities were unknown.

On admission, she was hypertensive, with a blood pressure of 187/68 mmHg, and had a low-grade fever of 100.2°F. Initial laboratory results were within normal limits.

Non-contrast CT of the head revealed hypodensity in the right parasagittal frontal lobe with loss of gray-white differentiation and mild swelling, concerning for acute or subacute infarct.

**Figure 1:**
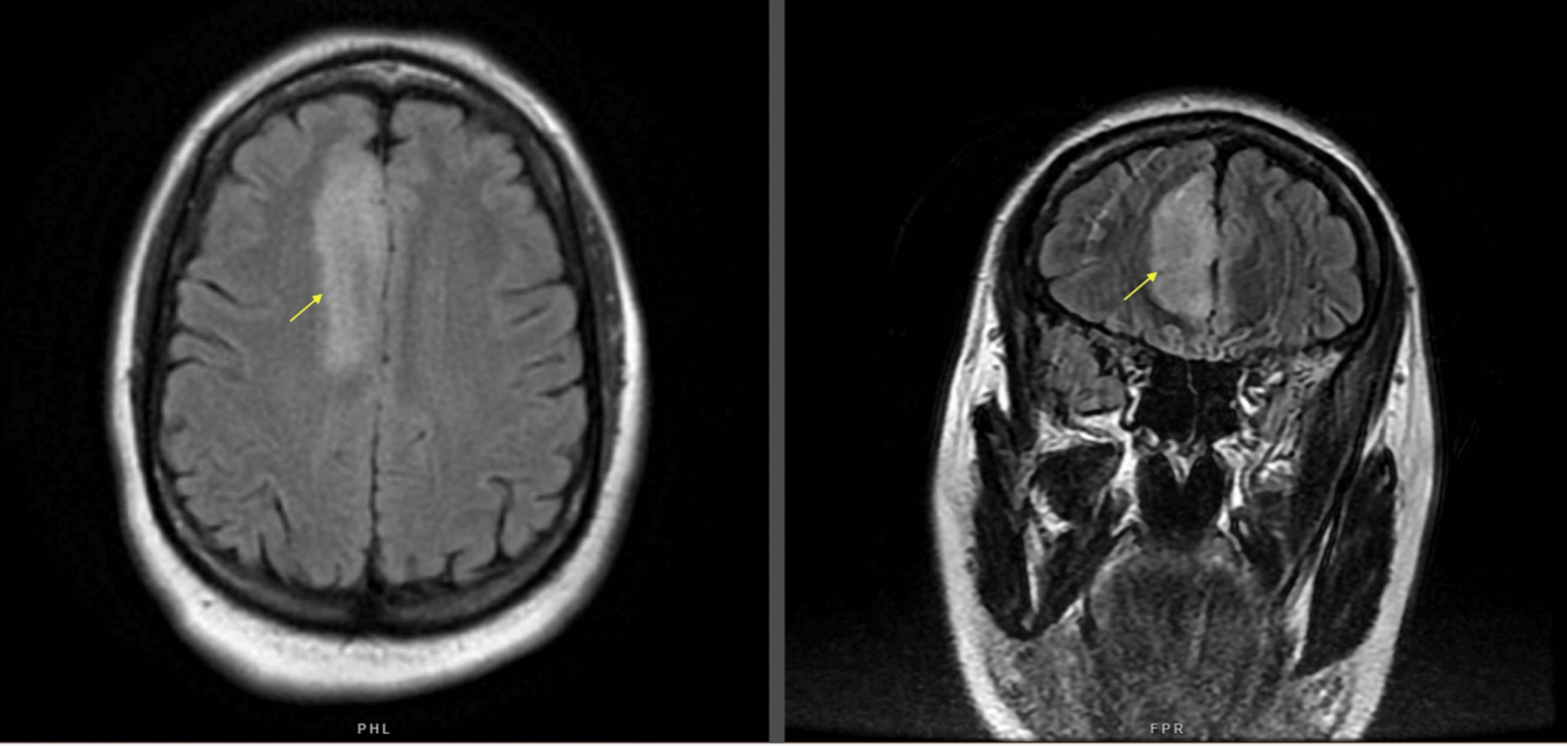
(A) MRI of the brain without IV contrast, axial view, T2 FLAIR PROPELLER, and (B) coronal view, T2 FLAIR, showing a moderate-sized area of restricted diffusion in the right parasagittal frontal lobe, compatible with an acute or subacute infarct located in the right anterior cerebral artery distribution. There is associated edema, swelling, and local mass effect, with sulcal effacement and slight right-to-left deviation of the frontal lobe by approximately 2 mm. No other areas of acute infarction are seen. Yellow arrows indicate the area of restricted diffusion corresponding to the infarct. FLAIR: Fluid-attenuated inversion recovery.

Brain MRI without IV contrast was performed using T1-weighted, T2-weighted, inversion recovery, gradient echo, and diffusion-weighted techniques. Imaging demonstrated a moderate-sized area of restricted diffusion in the right parasagittal frontal lobe within the anterior cerebral artery distribution, compatible with acute-to-subacute infarction. There was associated edema, sulcal effacement, and approximately 2 mm of midline shift (Figure 1).

Due to the duration of symptoms, she was not a candidate for thrombolytic therapy or mechanical thrombectomy. Medical management was initiated.

**Figure 2:**
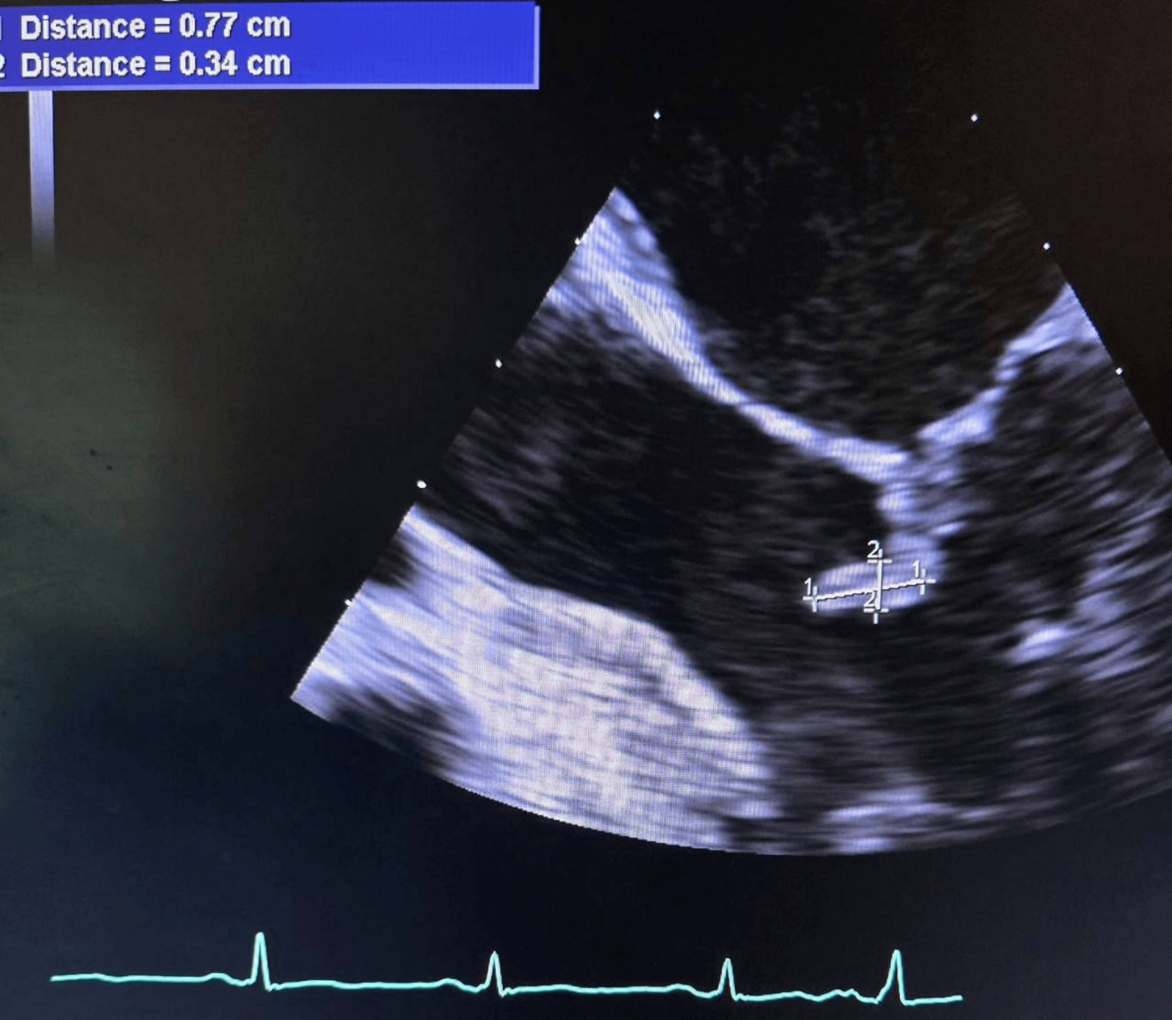
Echocardiographic image demonstrating mitral valve echodensity concerning for vegetation. Representative echocardiographic image demonstrating an echodensity attached to the mitral valve and concerning for vegetation. The lesion measured approximately 0.77 × 0.34 cm on echocardiographic assessment.

A transthoracic echocardiogram (TTE) revealed a mildly thickened mitral valve, mild regurgitation, ruptured chordae, and a possible vegetation (Figure 2). A transesophageal echocardiogram (TEE) confirmed a flail mitral valve leaflet with an echodensity measuring approximately 0.77 × 0.34 cm, concerning for vegetation.

Three sets of blood cultures were obtained on admission and remained negative. Several hours after TEE, the patient developed a fever of 102.5°F, prompting repeat blood cultures, which subsequently grew *Proteus mirabilis* in all four blood culture bottles. Given the patient's history of opioid use disorder, evaluation for active injection drug use was performed on admission. The patient denied recent IV drug use, reporting that her last use of cocaine and heroin had been approximately one year earlier via the intranasal route. Urine toxicology screening obtained on admission was negative for cocaine, opiates, oxycodone, cannabinoids, benzodiazepines, barbiturates, amphetamines, and phencyclidine.

Urine and respiratory cultures were negative. Computed tomography angiography (CTA) of the chest and CT of the abdomen and pelvis revealed no acute findings except for fecal loading. No additional source of bacteremia was identified during the diagnostic evaluation.

Initially, the cause of stroke was attributed to large-vessel atherosclerosis or a cardioembolic source. The detection of mitral valve vegetation and subsequent positive blood cultures supported a diagnosis of IE complicated by cardioembolic stroke. The absence of urinary, intestinal, or respiratory symptoms made the identification of a primary source challenging.

The patient received aspirin 324 mg orally in the ED and was subsequently transitioned to aspirin 81 mg, clopidogrel 75 mg daily, and atorvastatin 40 mg. IV ceftriaxone, 2 g daily, was initiated once *Proteus mirabilis* bacteremia was confirmed.

The patient completed a six-week course of IV ceftriaxone at a skilled nursing facility. Repeat blood cultures obtained after initiation of antimicrobial therapy demonstrated no growth, confirming microbiologic clearance of bacteremia. At the three-month follow-up, she reported stable health without systemic symptoms. Mild residual weakness in the left thigh persisted, attributed to the embolic infarct, and she was referred to physical therapy. She denied fever, chills, chest pain, or genitourinary symptoms. Follow-up TTE demonstrated persistent subvalvular echogenicity with moderate mitral regurgitation, likely representing fibrotic sequelae rather than active infection.

## Discussion

Non-HACEK Gram-negative IE remains an uncommon but clinically significant entity due to its association with delayed diagnosis, embolic complications, and limited evidence to guide management [1,2].

The 2023 Duke-International Society for Cardiovascular Infectious Diseases (Duke-ISCVID) criteria incorporate updated microbiologic and imaging considerations to improve diagnostic sensitivity in atypical or subacute presentations [4]. Our patient fulfilled the criteria for definite IE based on one major criterion, echocardiographic evidence of mitral valve vegetation, and four minor criteria: fever, a prior history of IE, a vascular phenomenon, ischemic stroke, and microbiologic evidence, defined as positive blood cultures for *Proteus mirabilis* not meeting the major microbiologic criterion. Although the initial blood cultures obtained on admission were negative, repeat blood cultures obtained after the onset of fever yielded high-grade *Proteus mirabilis* bacteremia, fulfilling the microbiologic minor criterion. This diagnostic sequence illustrates the importance of interpreting microbiologic findings together with echocardiographic and clinical features when evaluating suspected IE [4]. *Proteus mirabilis* remains an exceptionally rare cause of native valve IE [3].

Existing literature consists primarily of isolated case reports and small series, often involving patients with genitourinary infection, structural heart disease, or substance use [3]. A 2020 systematic review identified only 16 reported cases of *Proteus* IE between 1950 and 2019. In that review, concurrent urinary tract infection was reported in seven of 16 patients, the mitral valve was commonly involved, and mortality exceeded 30% [3]. Our case similarly involved the native mitral valve but differed in the absence of an identifiable primary source of bacteremia despite extensive diagnostic evaluation.

An important feature of this case was the coexistence of ischemic stroke, mitral valve vegetation, and subsequent *Proteus mirabilis* bacteremia. Although a definitive causal relationship between the stroke and IE cannot be established, septic embolism was considered the most likely etiology given the echocardiographic evidence of mitral valve vegetation and fulfillment of the 2023 Duke-ISCVID criteria for definite IE [4,5]. Non-contrast brain MRI demonstrated no findings suggestive of an intracranial abscess or other focal infectious complications. Although contrast-enhanced MRI is more sensitive for detecting intracranial abscesses, it was not performed in this case.

Current American and European guidelines recommend prolonged IV antibiotic therapy for non-HACEK Gram-negative IE; however, organism-specific recommendations for *Proteus* IE are lacking because of the rarity of these infections [1,2]. In our case, susceptibility-directed ceftriaxone monotherapy was selected based on organism identification, antimicrobial susceptibility results, and the absence of detected resistance markers on rapid molecular testing. Surgery was not pursued, as there was no evidence of heart failure, persistent bacteremia, large vegetation, or other guideline-based indications for early surgery. Our treatment approach was informed by previously reported cases summarized by Ioannou et al., while recognizing that no standardized regimen exists for this rare entity [3]. Although our patient responded favorably to ceftriaxone monotherapy, broader conclusions regarding the optimal treatment of *Proteus* IE cannot be drawn from a single case.

## Conclusions

*Proteus mirabilis* is a rare cause of native valve IE and may present subacutely, with ischemic stroke as an initial manifestation. This case illustrates one possible presentation of *Proteus mirabilis* IE, highlighting the importance of integrating microbiologic, echocardiographic, and clinical findings when evaluating patients with stroke of unclear etiology. Although no identifiable primary infectious source was found in our patient, broader conclusions regarding the clinical presentation, diagnostic approach, or optimal management of *Proteus* IE cannot be drawn from a single case.
